# A significant cancer burden and high mortality of intrahepatic cholangiocarcinoma in Thailand: a nationwide database study

**DOI:** 10.1186/s12876-016-0565-6

**Published:** 2017-01-05

**Authors:** Sombat Treeprasertsuk, Kittiyod Poovorawan, Ngamphol Soonthornworasiri, Roongruedee Chaiteerakij, Kessarin Thanapirom, Pisaln Mairiang, Kookwan Sawadpanich, Kanokwan Sonsiri, Varocha Mahachai, Kamthorn Phaosawasdi

**Affiliations:** 1 Faculty of Medicine, Division of Gastroenterology, Chulalongkorn University, Patumwan, Bangkok Thailand; 2 Thai Red Cross, Pathumwan, Bangkok Thailand; 3 Department of Clinical Tropical Medicine, Faculty of Tropical Medicine, Mahidol University, Ratchathewi, Bangkok Thailand; 4 Department of Tropical Hygiene, Faculty of Tropical Medicine, Mahidol University, Ratchathewi, Bangkok Thailand; 5 Faculty of Medicine, KhonKaen University, Muang District, KhonKaen Thailand; 6 Medicine, Vichaiyut hospital and Medical Center, Ratchathewi, Bangkok Thailand; 7 Department of Medicine, Faculty of Medicine, Chulalongkorn University, Rama 4 Road, Pathumwan District, Bangkok 10330 Thailand

**Keywords:** Intrahepatic cholangiocarcinoma, Cancer burden, Thailand, Nationwide database

## Abstract

**Background:**

We aimed to examine the burden of intrahepatic cholangiocarcinoma (ICC) in Thailand and identify the prognostic factors for all-causes of death.

**Methods:**

We conducted a population-based study of ICC patients admitted during 2009–2013 using the Nationwide Hospital Admission Database, the National Health Security Office (NHSO). There was an average of 1,051,146 patients/year with diagnosis of gastrointestinal diseases (GI). All patients with a diagnosis of ICC (ICD10- C221) were included from a total of 72,479 admissions from 858 hospitals. The surgical resection procedures such as the radical pancreaticoduodenectomy, subtotal and partial hepatectomy were analyzed. Data for all patients were censored 1 year post-study or death, whichever came first.

**Results:**

A total of 34,325 patients with ICC during a 5-year study period (on average, 6865 patients/year, with the incidence rate of 14.6 per 100,000 population, per year. The ICC patients had a mean age of 63.8+/−11.6 years and 63% were males. The mean length of hospital stay was 6.4+/−7.3 days with a mean+/−SD cost of hospitalization of $595+/−$1160 USD per admission. There were 659 patients (1.9%) underwent surgical resection. The overall survival of ICC patients with surgery was significantly better than those patients without surgery. Hazard ratio of death for patients without surgery was 2.5 (95% CI of 2.3–2.7). Approximately 14% of the ICC patients died during hospitalization. The median overall survival of all patients after the first admission was 53 +/−0.6 days. From the multivariate analysis, factors related to all-causes of death were: patients’ age >60 years (OR = 1.2, 95% CI; 1.1–1.3), length of hospital stay of >7 days (OR = 1.1, 95% CI; 1.02–1.2), male (OR = 1.3, 95% CI; 1.2–1.4), living in the northern part of Thailand (OR = 1.5, 95% CI; 1.3–1.8) and presence of complications during admission (OR = 1.3, 95% CI; 1.1–1.5).

**Conclusion:**

The disease burden of patients with ICC in Thailand is significant with the incidence rate of 14.6 per 100,000 population, per year during 2009–2013 and showed high mortality rate of 14%.

**Electronic supplementary material:**

The online version of this article (doi:10.1186/s12876-016-0565-6) contains supplementary material, which is available to authorized users.

## Background

Cholangiocarcinoma is one of the major liver cancers in Asia caused by parasites such as *Opisthorchis viverrini* and *Clonorchis sinensis* [1]. In 2008, it has been reported that the relative risk for cholangiocarcinoma with *O viverrini* is 7.7 and is prevalent in Southeast Asian countries [2]. Other contributing risk factors are hepatolithiasis, primary sclerosing cholangitis, chronic viral hepatitis B and C, cirrhosis, diabetes, obesity and Caroli’s disease [3]. Thailand has reported a high incidence of cholangiocarcinoma, especially in the KhonKaen Province, and is more prevalent in men than in women with an incidence of 84.6 vs. 36.8 per 100,000 persons per year, respectively [4].

The current staging of cholangiocarcinoma is based on the anatomical obstruction and location, and divided into 2 groups: extrahepatic (80%) and intrahepatic cholangiocarcinoma (20%) [3]. Intrahepatic cholangiocarcinoma (ICC) was classified and prognosed according to the number of tumors present, its differentiation, lymph node metastases and vascular invasion [5] whereas extrahepatic cholangiocarcinoma is classified as perihilar cholangiocarcinoma, Klatskin tumor or distal common bile duct cholangiocarcinoma [6] Recently, the Mayo clinic group proposed a new staging system based on the size and number of the tumor, vascular invasion and performance status which also showed that the median survivals of patients with perihilar-cholangiocarcinoma stages 1–4 varied from 48, 22, 8.6 and 3 months respectively [6]. This new Mayo clinical staging system is better than the TNM classification system which was based on histopathological findings in predicting the survival outcome of patients with perihilar cholangiocarcinoma [3, 6].

Current and novel therapies, including surgical or endoscopic treatment with neoadjuvant chemo-radiation therapy, may improve the survival in patients with certain cholangiocarcinoma stages [5, 7]. The intrahepatic cholangiocarcinoma (ICC) had the highest incidence in Asia [8], however the definite cause remains unclear. Additionally, ICC is associated with a high rate of fatality because of its nature of early invasion, widespread metastasis, and lack of effective treatment [9]. The median survival for Thai patients with advanced stages of cholangiocarcinoma was 5–6 months with palliative care [10]. However, cholangiocarcinoma patients with stages 3–4 had a higher mortality rate than those with stages 1–2 by 6.8 folds [11]. Yet there is a lack of data on the burden and outcome of ICC from the country with high prevalence of disease. The data reported from the previous studies were obtained from different hospital-based databases so the authors worked with the government to examine the burden of ICC in the country, and identify the prognostic factors for all causes of death of patients with ICC.

## Methods

### Study design

Thailand adopted a universal public health care system in 2002, which is comprised of three national health insurance categories: Civil Servant Medical Benefit Scheme (CSMBS), Social Security Scheme (SSS) and Medical Welfare Scheme (MWFS). CSMBS includes all civil servant officers with close family which individually reimburses medical expenses from the Controller General’s Department. SSS includes all Thai employees who are registered to the National Social Security Fund. MWFS includes all the remaining Thai citizens in the national database, which can access public health services from public hospitals and private hospital registered with The National Health Security Office (NHSO). NHSO database is the data from main universal public health care system in Thailand since 2002 which includes more than 80% of the population and more than 90% of all admissions from most public hospitals and some private hospitals in Thailand. Therefore, we conducted a population-based cohort study of ICC patients in Medical Welfare Scheme from 2009 to 2013 using the government’s health care database, the NHSO, Thailand. There was an average of 1,051,146 patients/year or 1,365,557 admissions/year with diagnosis of gastrointestinal diseases (GI). All patients with a diagnosis of ICC (ICD10- C221) were included from a total of 72,479 admissions from 858 hospitals. All patients were assessing the survival status until September 2014 using death registration.

### Study population

All ICC patients included in the study were more than 19 years old. The patient baseline characteristics and clinical outcomes were obtained from the patients’ main health insurance schemes of Medical Welfare Scheme. From the NHSO database, which mainly focus on the cost of hospitalization based on the diagnosis or diagnosis-related group (DRG). We extracted the following variables: the number of hospitalizations, mortality rate, complications, length of hospital stay, co-morbidity and the medical expenses per hospitalization.

### Inclusion criteria

From the annual rate of the overall hospitalization of 5,658,937 admissions, there was an average of 1,365,557 admissions per year from GI diseases which accounted for 24% of the overall hospitalization annually. We included all patients with primary diagnosis of ICC from 72,479 admissions during the 5-year period. (The details of the total annual hospitalization and the number of hospitalization from GI diseases based on ICD codes were reported in Additional file 1: Tables S1–S2).

The reported frequency of ICC for 2009 was 15.9%, for 2010 was 19.2%, for 2011 was 19.4%, for 2012 was 21.8% and for 2013 was 23.8%. The NHSO provided us access to the data from the Nationwide Hospital Admission Database.

### Definitions

The diagnosis of ICC, co-morbidities and complications were based on the International Statistical Classification of Diseases and Related Health Problems 10th Revision (ICD-10).

Co-morbidities and complications were identified by the following medical codes: coronary artery heart disease (CAD)-I251; congestive heart failure (CHF)-I500; chronic kidney disease (CKD) stages 1–5 -N181-N185; chronic obstructive pulmonary disease (COPD), unspecified-J449; history of stroke, not specified as hemorrhage or infarction-I64; diabetes mellitus (DM)- E149; sepsis- A419; ICD9 procedure; renal failure requiring dialysis-PRO3995; respiratory failure requiring ventilation for less than 96 consecutive hours-PRO9671; respiratory failure requiring ventilation for more than 96 consecutive hours-PRO9672; and infected wound surgical-T814.

Operable case with ICC was defined as a patient who underwent surgical resection procedure such as radical pancreaticoduodenectomy (procedure ICD9 CM 527), subtotal (procedure ICD9 CM 504) or partial hepatectomy (procedure ICD9 CM 5022).

The outcome measurements were the mortality rate, the proportion of complications, the length of hospital stay and the factors influencing mortality rate. Death was defined as all-causes of death from 2009 to September 28, 2014.

### Statistical analyses

The patients were categorized into 2 groups according to their treatment options: surgical and non-surgical groups. Patients in the surgical group were operable cases and defined as those ICC patients who underwent the surgical resection procedures such as radical pancreaticoduodenectomy, subtotal or partial hepatectomy. For the non-surgical group, these patients received non-surgical treatment. The differences between the 2 groups were compared by using student t test for continuous variables and were tested by the Chi-square test for proportions. Continuous outcomes are presented as the mean ± standard deviation (SD), and categorical data are presented as numbers (percentage). In this study, two-sided *P-* values <0.05 were considered statistically significant. This study used the SPSS statistical software package (SPSS Version 13) for analysis. The study was approved by the Institutional Review Board (IRB 113/58; Apr27, 2015) of the Faculty of Medicine, Chulalongkorn University, Bangkok, Thailand.

## Results

### Baseline characteristics and the burden of ICC in Thailand

From a total of 72,479 hospitalization with diagnosis of ICC based on ICD-221, there were 34,325 patients during the 5-year study period (on average, 6865 patients/year). The number of registration of Thai people in the NHSO system comprise of 47,000,000 people which is the majority group (67.6%) of the population of Thailand in 2013 which is 69.52 million people. Based on this number, the incidence rate of 14.6 per 100,000 population, per year (details in Additional file 1: Table S2). The mean age was 63.8+/−11.6 years. 63% were males. Overall cases of ICC were geographically distributed nationwide with predominant in northeastern part Thailand. The baseline characteristics and their comorbidities are shown in Table 1. The mean length of hospital stay was 5.7+/−6.9 days with a mean+/−SD cost of hospitalization of US$595+/−1160 USD per admission (exchange rate 35.62 baht/US dollar).

**Table 1:** The baseline characteristics and comorbidities of the overall admissions of ICC patients from 2009 to 2013

Variables	ICC patients (*n* = 72,749 admission)
Age (mean+/− SD)	63.79 +/−11.59
- Age >60-year, *n* (%)	61.6%
Sex (%male)	63.2%
Hospital (H) level; %	
- Community H.	76.6%
- General H.	14.5%
- Tertiary care H.	5.1%
- Private H.	0.9%
- others (NHSO, no data)	2.9%
Residence by region; %	
- Central	9.8%
- Northeast	60.2%
- North	22.5%
- South	1.6%
- others	6.0%
Comorbidities; %	
- Atherosclerotic heart disease	0.1%
- Congestive heart failure	0.5%
- COPD	0.9%
- Diabetes Mellitus	0.2%
- Stroke	0.1%
- Chronic kidney disease	0.4%
- Cirrhosis	2.7%
- Viral Hepatitis B	0.4%
- Viral Hepatitis C	0.3%
- HIV infection	0.2%

Note: *COPD* chronic obstructive pulmonary disease

### Treatment outcomes and prediction of mortality

**Fig. 1:**
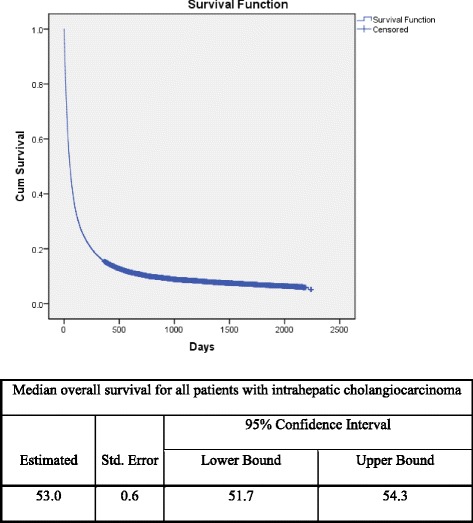
Mean overall survival of all patients with intrahepatic cholangiocarcinoma after first admission

**Fig. 2:**
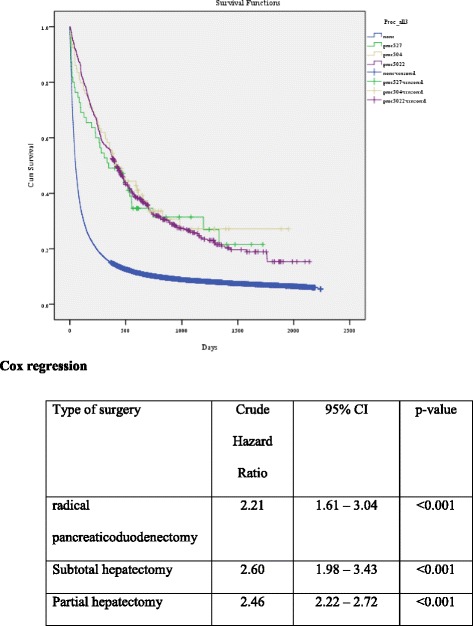
Mean overall survival for all patients with intrahepatic cholangiocarcinoma with surgery compared to those without surgery

There were 659 ICC patients (1.9%) who had resectioned surgery such as radical pancreaticoduodenectomy (*n* = 55), subtotal (*n* = 79) or partial hepatectomy (*n* = 525). Treatment outcomes for patients with surgery were significantly better than those without surgery are shown in Fig. 1. The overall survival of patients with surgery was significantly better than those patients without surgery. Figure 2 and Table 2 show the hazard ratio of death for patients without surgery which was 2.5 (95% CI of 2.3–2.7). There was no significant difference of survival time among the 3 different procedures (radical pancreaticoduodenectomy, subtotal or partial hepatectomy).

**Table 2:** The survival outcome of patients with intrahepatic cholangiocarcinoma classified by the type of surgical treatment

	Univariate Cox regression		
Type of surgery	Crude hazard ratio	95% CI	*p*-value
Radical pancreaticoduodenectomy	2.2	1.61–3.04	<0.001
Subtotal hepatectomy.	2.6	1.98–3.43	<0.001
Partial hepatectomy.	2.5	2.22–2.72	<0.001
Presence of any one of three surgical treatment options	2.5	2.25–2.70	<0.001

From 34,325 patients ICC patients, 4777 patients died during the follow-up period. The all-cause mortality rate was 14%. The median overall survival for all patients after first admission was 53 +/−0.6 days (95% CI 51.7–54.3). The hazard ratio of death in patients without surgery was 2.5 (95% CI of 2.3–2.7). From the multivariate analysis, factors related to all causes of death were the patients’ age >60 years (OR = 1.2, 95% CI; 1.1–1.3), length of hospital stay (LOS) of >7 days (OR = 1.1, 95% CI; 1.02–1.2), male (OR = 1.3, 95% CI; 1.2–1.4), living in the northern part of Thailand (OR = 1.5, 95% CI; 1.3–1.8) and presence of complications during admission (OR = 1.3, 95% CI; 1.1-1.5). In addition, we found that the ICC patients who had LOS ≤7 days had significantly lower frequency of comorbidities including atherosclerotic heart disease , congestive heart failure, cirrhosis, viral hepatitis B viral hepatitis C and HIV infection than those with LOS >7 days (*p* <0.05) (Additional file 1: Table S3).

## Discussion

In the current study, the incidence rate of 14.6 per 100,000 population, per year with mortality rate of 14%. This NHSO database is nationwide study, and its coverage of at least of 67.6% of the population of Thailand in 2013 and this database were used and validate in several studies including disease burden of cirrhosis, drug induced liver injury and liver abscess [12–14]. Most of ICC patients presented with advanced stages by their clinical findings and complications. Due to the limited information of clinical staging, however in our study we had only 1.9% of ICC patients underwent surgical treatment. The new classification of advanced stage cholangiocarcinoma by the Mayo clinic group [6] mentioned that the TNM staging system is used to predict the disease outcome, which is mainly based on the histopathological information from the operation. Thus, the TNM staging cannot applied to the patients with ICC because most of them have unresectable disease which is supported by our data and it was similar to previous studies from Thailand [10, 11, 15, 16]. Our study demonstrated that several factors predicted poor survival outcome of hospitalized ICC patients included advanced age >60 years, length of hospital stay of >7 days which related to the more frequency of comorbidities, male, living in the northern part of Thailand, and presence of complications during admission. In addition, the tumor size of at least 5 cm and the regional lymph node metastasis were reported as key predictors of higher chance of perineural or vacular invasion [17]. This important information regarding to the ICC characteristics including the size of tumor and histopathology were lacking in our database thus we cannot add this variables into our analysis. One of the major reason of poor outcomes of ICC patients in Thailand is the lacking of early diagnosis. The heterogeneous of tumor biology, risk factors and clinical features may be the reasons for the difficulty of developing surveillance measures in high risk group [18, 19]. Our data of disease burden of ICC with high mortality rate of 14%. as well as low proportion of ICC patients underwent surgical treatment (1.9%) may be useful for the health care policy makers and the clinicians to consider for implement health care policy or setting up the surveillance measures in other centers.

The main strength of this study is the fact that we had access to the nation’s health care database, Nationwide Hospital Admission Database, so was able to assess the disease burden of ICC with the incidence rate of 14.6 per 100,000 population, per year during 2009–2013. Even though, there is limited information in clinical findings but the burden of intrahepatic cholangiocarcinoma which has high mortality rate and most of them present as non-operable stage are valuable for national health care policy planning for early stage of cancer detection. The average costs of hospitalization of the GI and liver related diseases in Thailand during 2009–2013 are lower than those reported in the United States [20] (Additional file 1: Table S4). However, there are some limitations. First, there was not enough information in regards to the major risk factors and staging of ICC development, such as liver flukes. This limitation was explained by the design of the nationwide database which focused on financial purpose of reimbursement, no for clinical data collection. Second, the parameters outside of the Nationwide Hospital Admission Database including the clinical information, the blood tests were not collected.

## Conclusions

The disease burden of patients with ICC in Thailand is significant with the incidence rate of 14.6 per 100,000 population, per year and showed high mortality rate of 14%. The health promotion and prevention as well as the early stage of ICC detection may be a relevant strategy in developing countries.

## Additional file

Additional file 1: Table S1. The Nationwide Hospital Admission Data, the National Health Security Office (NHSO), Thailand, showed an average of 1,051,146 patients/year or 1,365,557 admissions/year with diagnosis of gastrointestinal diseases (GI) during 2009–2013. **Table S2** Hospitalization patients with diagnosis of ICC based on ICD-221 (*N* = 72,479 admissions) during the 5-year period. **Table S3.** Comparison ICC patients who had length of hospital stay (LOS) ≤7 days and those with LOS >7 days. **Table S4.** The average cost of hospitalization and the in-hospital mortality rate classified by disease; during 2009–2013 from the Nationwide Hospital Admission Data of Thailand. (DOCX 20 kb)

## Abbreviations

CAD: Coronary artery heart disease
CHF: Congestive heart failure
CKD: Chronic kidney disease
COPD: Chronic obstructive pulmonary disease
DM: Diabetes mellitus
GI: Gastrointestinal diseases
ICC: Intrahepatic cholangiocarcinoma
ICD: International Statistical Classification of Diseases and Related Health Problems
NHSO: National Health Security Office
SD: Standard deviation
UC: Universal coverage
