# Supplementary material for: A significant cancer burden and high mortality of intrahepatic cholangiocarcinoma in Thailand: a nationwide database study
Source: BMC Gastroenterol. 2017 Jan 5;17:3. doi: 10.1186/s12876-016-0565-6 (PMC5216607; doi:10.1186/s12876-016-0565-6)
Supplement: Additional file 1: Table S1. — The Nationwide Hospital Admission Data, the National Health Security Office (NHSO), Thailand, showed an average of 1,051,146 patients/year or 1,365,557 admissions/year with diagnosis of gastrointestinal diseases (GI) during 2009–2013. Table S2 Hospitalization patients with diagnosis of ICC based on ICD-221 (N = 72,479 admissions) during the 5-year period. Table S3. Comparison ICC patients who had length of hospital stay (LOS) ≤7 days and those with LOS >7 days. Table S4. The average cost of hospitalization and the in-hospital mortality rate classified by disease; during 2009–2013 from the Nationwide Hospital Admission Data of Thailand. (DOCX 20 kb) [file 12876_2016_565_MOESM1_ESM.docx]

**Additional file 1**

**Table S1** The Nationwide Hospital Admission Data, the National Health Security Office (NHSO), Thailand, showed an average of 1,051,146 patients/year or 1,365,557 admissions/year with diagnosis of gastrointestinal diseases (GI) during 2009-2013.

|  | Average/year | 2009 | 2010 | 2011 | 2012 | 2013 |
| --- | --- | --- | --- | --- | --- | --- |
| Number of total admission per year | 5,658,937 | 5,387,774 | 5,680,852 | 5,624,263 | 5,733,702 | 5,868,094 |
| Number of admissions due to GI diseases (% of total admission) | 1,365,557  (24.1%) | 1,323,028  (24.6%) | 1,355,683  (23.8%) | 1,369,119  (24.3%) | 1,392,344  (24.3%) | 1,387,610  (23.6%) |
| No. of admitted patients with GI diseases | 1,051,146 | 1,033,820 | 1,048,244 | 1,054,070 | 1,067,312 | 1,052,286 |

**Table S2** Hospitalization patients with diagnosis of ICC based on ICD-221 (N = 72,479 admissions) during the 5-year period**.**

|  | Average/year | 2009 | 2010 | 2011 | 2012 | 2013 |
| --- | --- | --- | --- | --- | --- | --- |
| Number of admissions due to GI diseases (% of total admission) | 1,365,557 | 1,323,028 | 1,355,683 | 1,369,119 | 1,392,344 | 1,387,610 |
| Number of admissions due to ICC (% of GI admission) | 14,496  (1.06%) | 11,523  (0.87%) | 13,883  (1.02%) | 14,060  (1.03%) | 15,769  (1.13%) | 17,244  (1.24%) |
| Number of patients with ICC | 6865 | 6866 | 6630 | 6816 | 6986 | 7027 |

Note: The number of registration of Thai people in the NHSO system comprise of 47,000,000 people which is the majority group (67.6%) of the population of Thailand in 2013 which is 69.52 million people. Based on this number, the incidence rate of 14.6 per 100,000 population, per year

**Table S3** Comparison ICC patients who had length of hospital stay (LOS) <7 days and those with LOS >7 days

| **Comorbidities** | LOS (>7days)  (n=9,092) | LOS (<=7days)  (n=25,232) | P-value |
| --- | --- | --- | --- |
| Atherosclerotic heart disease | 20(0.22) | 29(0.11) | 0.023* |
| Congestive heart failure | 91(1.00) | 104(0.41) | <0.001* |
| COPD | 89(0.98) | 250(0.99) | 0.921 |
| Diabetes Mellitus | 19(0.21) | 35(0.14) | 0.147 |
| Stroke | 11(0.12) | 25(0.10) | 0.580 |
| Chronic kidney disease | 52(0.57) | 160(0.63) | 0.516 |
| Cirrhosis | 396(4.36) | 877(3.48) | <0.001* |
| Viral Hepatitis B | 78(0.86) | 127(0.50) | <0.001* |
| Viral Hepatitis C | 51(0.56) | 101(0.40) | 0.048* |
| HIV | 35(0.38) | 51(0.20) | 0.003 |

**Table S4** The average cost of hospitalization and the in-hospital mortality rate classified by disease; during 2009-2013 from the Nationwide Hospital Admission Data of Thailand.

| **Gastrointestinal diagnosis**  **based on ICD -10** | **Number of annual admissions (admission)** | **Average cost of hospitalization per admission (USD)*** | **In-hospital mortality rate (%)** | **Mean length of stay (days)** | **Burden of GI diseases and cost in the US:** median cost, USD** in 2012 |
| --- | --- | --- | --- | --- | --- |
| Acute diarrhea (A09) | 214,722 | 99±230 | 0.6% | 2.3 | 4070 (GI infection) |
| Peptic ulcer bleeding (K25.0-K28.0) | 10,838 | 666±1,210 | 3% | 4.9 | 6700 (GI hemorrhage) |
| Liver abscess (K75.0 and A06.4) | 11,296 | 846±1,574 | 2.8%. | 8.0 | No data to access |
| Toxic liver diseases (K71.0) | 6,516 | 69±110 | 3.2% | 5.0 | No data to access |
| Acute liver failure (K72.0 and K71.1) | 4,118 patients/year | 1,037 ± 2,679 | 18.4% | 8.7 | No data to access |
| Cirrhosis with complications (K 74.0) | 9, 649 | 1,177±1,938 | 26% | 8.6 | 38,223  (Ascites and SBP) |

**Note:** *exchange rate 35.62 baht/US dollar (USD) compared to the average expense per person per year in 2008 = 50 USD; ** Reference: Peery AF, Crockett SD, Barritt AS, et al.  **Burden** of Gastrointestinal, Liver, and Pancreatic Diseases in the United States. Gastroenterology. 2015 Dec;149(7):1731-1741.
